# CDK4/6 as a Therapeutic Target in HR+/HER2− Breast Cancer Cells—Current Treatment Status

**DOI:** 10.3390/cancers17061039

**Published:** 2025-03-20

**Authors:** Kamila Krupa, Anna Liszcz-Tymoszuk, Natalia Czerw, Aleksandra Czerw, Katarzyna Sygit, Remigiusz Kozłowski, Andrzej Deptała, Anna Badowska-Kozakiewicz

**Affiliations:** 1 Students’ Scientific Organization of Cancer Cell Biology, Department of Oncology Propaedeutics, Medical University of Warsaw, 01-445 Warsaw, Poland; aliszcz@hotmail.com (A.L.-T.); s082240@student.wum.edu.pl (N.C.); 2 Department of Health Economics and Medical Law, Medical University of Warsaw, 01-445 Warsaw, Poland; aczerw@pzh.gov.pl; 3 Department of Economic and System Analyses, National Institute of Public Health NIH—National Research Institute, 00-791 Warsaw, Poland; 4 Faculty of Health Sciences, Calisia University, 62-800 Kalisz, Poland; k.sygit@uniwersytetkaliski.edu.pl; 5 Department of Management and Logistics in Healthcare, Medical University of Lodz, 90-131 Lodz, Poland; remigiusz.kozlowski@umed.lodz.pl; 6 Department of Oncology Propaedeutics, Medical University of Warsaw, 01-445 Warsaw, Poland; andrzej.deptala@wum.edu.pl (A.D.); anna.badowska-kozakiewicz@wum.edu.pl (A.B.-K.)

**Keywords:** breast cancer, CDK4/6 inhibitors, palbociclib, ribociclib, abemaciclib, dalpiciclib, HR+/HER2−

## Abstract

Breast cancer is the most frequently diagnosed neoplasm in the world. It can be classified into four main subtypes, each of them showing differences in the expression of hormone receptor (HR), human epidermal growth factor receptor 2 (HER2), and in cell metabolism. Since 2015, when The U.S. Food and Drug Administration (FDA) approved the first cyclin-dependent kinase 4 and 6 (CDK4/6) inhibitor that regulates the cell cycle, treatment of HR+/HER2− BC has become much more effective. Currently, palbociclib, ribociclib, and abemaciclib are more often used both in combination with endocrine therapy as well as in monotherapy. Their application has been extensively verified in many clinical trials such as PALOMA-1,2,3, MONALEESA-1,2,3,7, and MONARCH-1,2,3, which allowed the verification of differences in their effectiveness, dosage, and adverse effects. Subsequent studies, MonarchE and NATALEE, examined the role of these inhibitors as adjuvant therapy, as well as at verifying their safety. Moreover, dalpiciclib is being investigated in HR+/HER2− BC treatment. This article will summarize clinical efficacy, recommendations, and differences in toxicity profile between palbociclib, ribociclib, and abemaciclib and will also discuss the possibility of using dalpiciclib in the treatment of breast cancer.

## Simple Summary

Breast cancer can be classified into four main subtypes based on hormone receptor (HR) and human epidermal growth factor receptor 2 (HER2) expression, which influence tumor metabolism and response to treatment. Cyclin-dependent kinase 4 and 6 (CDK4/6) inhibitors have significantly improved the treatment of HR+/HER2− breast cancer. The clinical effectiveness of palbociclib, ribociclib, and abemaciclib have been evaluated in trials such as PALOMA, MONALEESA, and MONARCH, which examined differences in efficacy, dosage, and side effects. Additionally, recent studies like MonarchE and NATALEE have investigated their potential in adjuvant settings. Furthermore, dalpiciclib is being studied in the treatment of HR+/HER2− breast cancer. This article provides an overview of the clinical applications, toxicity profiles, and future perspectives of CDK4/6 inhibitors.

## 1. Introduction

With more than 2.2 million new cases in 2020, breast cancer (BC) is the most frequently diagnosed neoplasm across the globe [1]. Over the last three decades, its incidence and death rates have significantly increased; for instance, the disease’s frequency more than doubled in 60 out of 102 nations between 1990 and 2016. Additionally, deaths have more than doubled in 43 out of 102 countries [2]. According to the surveillance, epidemiology, and end results (SEER) program, based on 2016–2020 data, the rate of new cases of female breast cancer was 126.9 per 100,000 women per year, where the median age at diagnosis was 63 years old [3]. Currently, people over 50 make up over 80% of BC patients [4].

Risk factors are categorized into two groups: modifiable and non-modifiable. The non-modifiable subgroup comprises female sex, older age, genetic mutations, family history of BC, race, previous history of BC or radiation therapy, pregnancy, and menopausal conditions [4,5,6]. Genetic mutation like BReast CAncer gene 1 (BRCA1) located on chromosome 17 and BReast CAncer gene 2 (BRCA2) located on chromosome 13 are strongly associated with an elevated risk of BC. Female carriers of these mutation have a lifetime risk of 50–85%, whereas for males it is 5–10% [7]. The modifiable subgroup involves hormonal replacement therapy, lack of physical activity, overweight, alcohol intake, a diet high in processed food and red meat, exposure to chemicals like iron, smoking, and excessive exposure to artificial light [4,5,6]. Furthermore, the modifiable risk factors are connected to each other. For instance, a diet rich in sodium, fat, and sugar, commonly found in ultra-processed food, predisposes individuals to overweight and obesity, which are established risk factors for BC [8].

## 2. Classification

Depending on the histological type, there are over 20 variants of BC invasive ductal carcinomas of no special type (IDC-NST) occurring most often. IDC-NST accounts for 40–75% of invasive forms of breast cancer. Invasive lobular carcinoma (ILC) is the second most frequently identified form of BC (approximately 5–15% of invasive forms of BC) [9]. Additional variations of BC include the mucinous, tubular, cribriform, micropapillary, papillary, medullary, metaplastic, and apocrine forms [10].

The expression levels of estrogen receptor (ER), progesterone receptor (PR), and human epidermal growth factor receptor 2 (HER2) and the number of cells containing them are useful predictors of response to hormonal therapy and allow the selection of appropriate treatment [6]. The HER2 gene is amplified in approximately 15–20% of BC [11], and its presence is associated with a more aggressive course of the disease and a shorter survival duration. On the other hand, ER and PR are expressed in 75% of tumors, and their appearance is associated with decreased aggression. Estrogens, by binding to the hormone receptors (HR), cause interaction with cyclin D and MYC, which may lead to cell growth. Excessive proliferation of the cancer cell may be contributed to by overexpression of HER2, leading to the activation of signaling pathways such as PI3K/AKT and MAPK [12]. Immunohistochemical (IHC) analysis of ER, PR, HER2, and Ki-67 (marker of proliferation Kiel 67) expression is used to categorize BC into four main molecular subtypes: Luminal A (HR+/HER2−), Luminal B (HR+/HER2−), HER2-enriched (HR−/HER2+), and basal-like [13,14,15]. The most commonly occurring BC subtype is the HR+/HER2− subtype, accounting for 74% of recorded cases. [16,17]. Table 1 shows the molecular types of BC along with the activity of ER, PR, HER2, and Ki-67 receptors in each of them.

## 3. Breast Cancer Cell Metabolism

The Luminal A and B subtypes of breast cancers are considered to have lower amino acid metabolism and lower glucose metabolism than HER-enriched and basal-like tumors. Luminal B exhibits higher glutamine metabolic activity than luminal A, which correlates with MYC activity. the HR−/HER+ phenotype displays higher amino acid, glutamine, and lipid metabolism than the others [18]. There is also the most aggressive form—triple negative breast cancer (TNBC), characterized by the absence of ER, PR, and HER2 receptor expression, which makes cancer cells unresponsive to endocrine therapy or HER2-targeted treatments [10,19]. It has only glucose metabolism activity with increased glycolysis and lactate production and low mitochondrial respiration (Table 1) [20,21].

## 4. Cell Cycle and Its Regulation

The cell cycle is an indispensable element of the cancer cell, and the discovery of its course and regulation has allowed the creation of new ways of treating cancer. The cell cycle consists of the interphase, which includes the G1, S, and G2 phases and mitosis (M phase). In the G1 and G2 phases, the cell prepares to divide, increasing its mass and volume. It also doubles organelles and synthesizes proteins necessary for division, such as tubulin. DNA replication occurs in the S phase, and cell division takes place in the M phase. In regular cells, division lasts approximately 24 h, with the interphase lasting 23 h and the M phase 1 h [22], while in cancer cells it may be shorter [23]. The transition from the G1 to S phase depends on conditions and signals from the environment, while the intracellular regulation during cell division is exercised by cyclins and cyclin-dependent kinases (CDKs) [24,25].

There are twenty cyclin-dependent kinases in the human body, but only six are directly involved in the cell cycle: CDK1-4 and CDK6-7. Others, such as CDK7-CDK11, participate in the regulation of gene expression [26]. CDK3, which binds cyclin C, is involved in the transition from the G0 to G1 phase and the phosphorylation of retinoblastoma protein (RB) [27,28]. Cyclins D1, D2, and D3 bind and activate the phosphorylation function of CDK4 and CDK6 while entering the cell cycle in the G1 phase, which is crucial for passing through the G1 phase [29,30].

**Figure 1:**
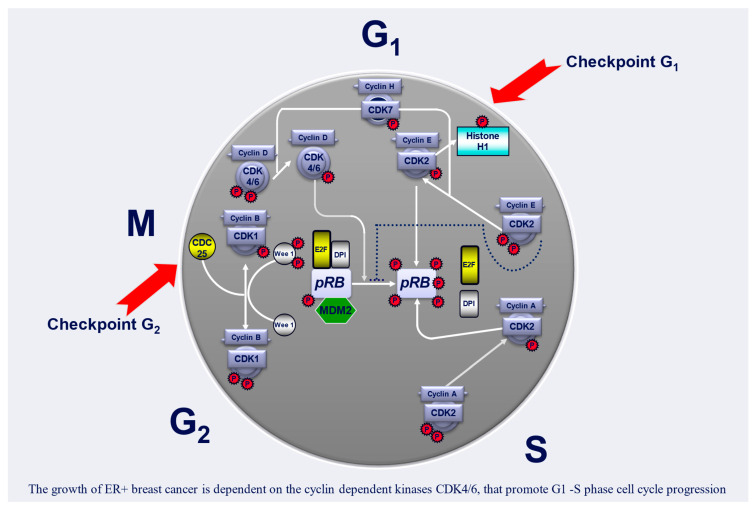
Simplified diagram of the regulation of the cell cycle (modified on Deptala A. habilitation thesis). Abbreviations: CDK—cyclin-dependent kinase; MDM2—mouse double minute 2 homolog; pRB—retinoblastoma protein; CDC25—cell division cycle phosphatase family; P—phosphorylation.

**Figure 2:**
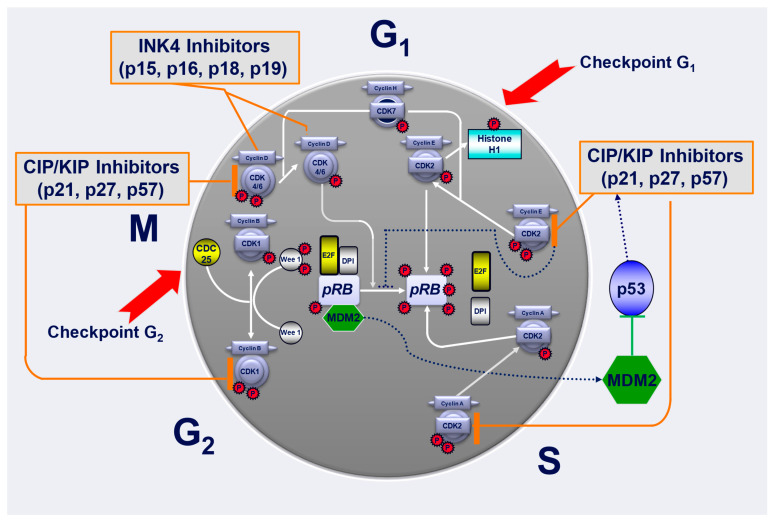
Simplified diagram of the regulation of the cell cycle including CIP/KIP and INK4 inhibitors (modified on Deptala A. habilitation thesis). Abbreviations: CDK—cyclin-dependent kinase; INK4—family of CDK inhibitors; CIP/KIPs—CDK-interacting proteins/kinase inhibitor proteins; MDM2—mouse double minute 2 homolog; pRB—retinoblastoma protein; CDC25—cell division cycle phosphatase family; P—phosphorylation.

CDK1 and CDK2 are engaged in the S and M phases. At the G1/S checkpoint, CDK2 binds to cyclin E, and after entering the S phase, the CDK2–CyclinA complex is formed. The S/G2 transition is mediated by the CDK1–CyclinA complex, and G2/M by CDK1–Cyclin B [28]. In healthy cells, extracellular signals, such as mitogens, lead to the synthesis of cyclin D and thus the stimulation of CDK4/6. The cell is directed to the G1 phase of the interphase. The CDK4/6–Cyclin D complex promotes the phosphorylation of the RB protein, which leads to its release from the inactive E2F-RB complex. E2F in its unbound form, as a transcription factor, leads to the expression of cyclin E, cyclin A, and cyclin B. They are required for further phases of the cell cycle (Figure 1) [26,31]. Cyclin-dependent kinase inhibitors (CDKIs) regulate the CDK–Cyclin complexes. This group includes the family of inhibitors of CDK4 and CDK6 proteins (INK4), namely p16, 15, p18, and p19, and CDK-interacting proteins/kinase inhibitor proteins (CIP/KIPs): p21, p27, and p57, which inactivate a broader range of CDK-cyclin complexes during the cell cycle (Figure 2) [26,32]. The E3 ubiquitin ligase also plays a significant role in regulating the expression of, among others, the Skp1–Cul1–F-box-protein (SCF) complex and anaphase promoting complex/cyclosome (APC/C), which control cell cycle transitions [33]. Another way to inhibit cell activity is targeted degradation of proteins (TPD), which causes its hyperactivity. There are three classes of compounds: single-ligand compounds that interact with the target protein, E3 ubiquitin ligase modulators, also called molecular glues, and proteolysis targeting chimera (PROTAC), where the E3 component and the selected protein are combined [24,34].

**Figure 3:**
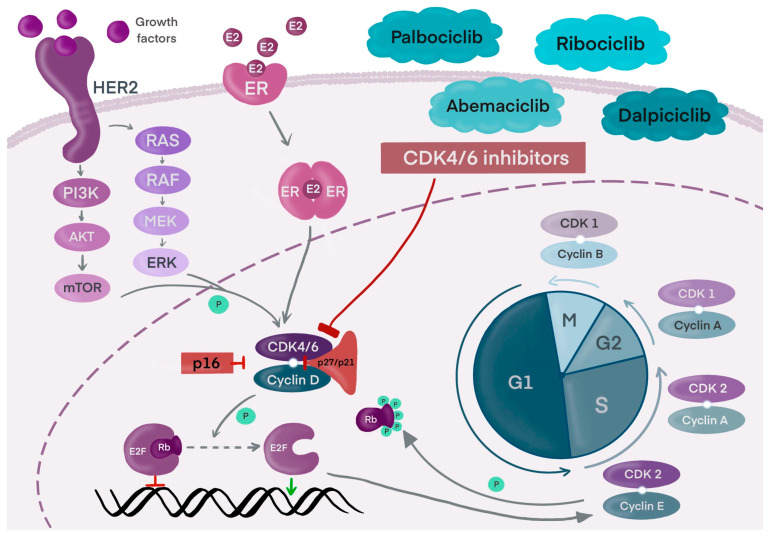
The mechanism of the signalization by HER2 and ER, cyclins, CDK, and other proteins related to cell division. CDK4/6 inhibitors regulating the cell cycle. Own work; based on [31]. Abbreviations: CDK—cyclin-dependent kinase; P—phosphorylation.

In cancer cells, cell cycle proteins are overactive, which leads to dysregulation and uncontrolled division. Modifications of their activity, through inhibition and degradation, may contribute to stabilizing the cell cycle and preventing carcinogenesis. Figure 3 shows the mechanism of action of cyclins, CDKs, and other proteins related to cell division.

## 5. Cyclin-Dependent Kinase 4 and 6 (CDK4/6) Inhibitors

Each of the molecular subtypes of BC displays distinction in modifying the cell cycle [33]. The lack of CIP/KIP and INK4 proteins along with the amplification of cyclin D1 and hyperactivation of CDK4/6 are the most commonly occurring changes [26]. The HR+/HER2− BC has been shown to have an increased level of expression of the estrogen receptor 1 gene (ESR1), and CCND1 gene (encodes cyclin D1), which results in cell cycle progression through phosphorylation and inactivation of the Rb protein. This allows the Rb to dissociate from E2F and enables it to function as a transcription factor [33,35]. Additionally, PIK3CA mutations are common in this subtype, leading to progression through increased AKT/mTOR signaling. TP53 mutations are rare in this subtype, which distinguishes it from HER2+ and TNBC breast cancers [36]. In the treatment of HR+/HER2− cancer, endocrine therapy (aromatase inhibitors or fulvestrant) reduces the synthesis of cyclin D1, while selective CDK4/6 inhibitors arrest the cell cycle [37]. In recent years, the Food and Drug Administration (FDA) has approved three CDK4/6 inhibitors, which are currently used in the treatment of breast cancer with a high recurrence risk [17]. These are cyclibs: palbociclib, ribociclib, and abemaciclib.

The Asp-Phe-Gly (DFG) tripeptide pattern composes the activation loop (A), which controls the substrate-binding active site of CDK. When the loop is phosphorylated, the kinase is activated. The DFG motif then adopts a position adjacent to the ATP binding site (DFG-in conformation), and if it is dephosphorylated, it blocks the binding site (DFG-out conformation) [24,38]. Although CDK4/6 inhibitors belong to the same group of first-type inhibitors, acting in DFG in conformation, they have slight differences in effectiveness and safety [24,39].

## 6. Common Terminology Criteria for Adverse Events (AEs)

Cancer patients encounter “untoward medical events” while participating in clinical trials, which are essential to evaluating the safety profile of the treatment. There are three distinct methods for categorizing AEs. A randomized trial’s treatment arms are usually compared using the frequency of grade 3 or worse AEs without considering the types of events. Another method is to assess the frequency of the worst grade of the most common AEs in the trial. The third option is to classify AEs into hematologic or non-hematologic groups [40]. Treatment with CDK4/6 inhibitors is associated with AEs like neutropenia, leukopenia, anemia, thrombocytopenia, or diarrhea. To assess the degree of organ toxicity and safety profile in patients receiving cancer therapy, the Common Terminology Criteria for Adverse Events (CTCAE) published by the National Cancer Institute (NCI) of the National Institutes of Health (NIH) are used [41] (Table 2).

## 7. Palbociclib

In 2015, the combination of letrozole and palbociclib was approved for the treatment ER+/HER2− advanced breast cancer (ABC), while in 2016, the FDA approved the use of palbociclib in combination with fulvestrant for the treatment of ABC or metastatic breast cancer (MBC) HR+/HER2− in women who progressed after hormonal therapy [42]. The results of the phase II clinical trial PALOMA-1 (NCT00721409) showed that progression-free survival (PFS) for the letrozole plus palbociclib group was increased compared to the letrozole alone group (20.2 months vs. 10.2 months; HR 0.488, 95% CI 0.319–0.748; one-sided *p* = 0.0004) [43,44]. The PALOMA-2 trial (NCT01740427), including 666 patients, of whom 444 received at least one dose of letrozole with palbociclib, confirmed the efficacy and safety of the combination [45,46]. This led to FDA approval of the palbociclib plus aromatase inhibitor (AI) combination in 2017 for postmenopausal women with HR+/HER2− ABC or MBC [42]. In the PALOMA-1 study, no febrile neutropenia was observed, but the incidence of neutropenia and leukopenia were increased. Grade 3 and 4 neutropenia was observed in 59% of patients in the palbociclib plus letrozole group compared to 1% of patients in the letrozole group, and leukopenia was observed in 18% compared to 0% of patients in the letrozole group. Other adverse events reported more frequently in the palbociclib plus letrozole group were fatigue, anemia, diarrhea, nausea, arthralgia, and thrombocytopenia [47]. Similar AEs were reported by patients in the PALOMA-2 trial, where neutropenia was the most frequent (82.2% vs. 6.3% with placebo plus letrozole) [48,49].

According to the phase III PALOMA-3 trial (NCT01942135), including patients with HR+/HER2− ABC whose disease had progressed after prior endocrine therapy (ET), the median PFS was 9.2 months for the fulvestrant plus palbociclib group compared to 3.8 months in the fulvestrant plus placebo group (HR 0.42, 95% CI 0.32–0.56; *p* < 0.001) [50,51]. Additionally, the risk of death was 28% lower in the group of patients who had never received chemotherapy palbociclib than in the group that received fulvestrant with placebo (HR 0.72, 95% CI 0.55–0.94; *p* = 0.008) [52]. Among AEs, neutropenia was ranked first, and leukopenia was ranked second [50]. Over six years of follow-up in patients with HR+/HER2− ABC a clinically meaningful improvement in OS linked to palbociclib with fulvestrant was maintained, although it was not statistically significant [53]. Breast cancers are relatively rare in men, accounting for less than 1% of all BC [54]; however, treatment with CDK4/6 should be tested. The POLARIS study (NCT03280303) showed that, in the group of male patients with HR+/HER2− ABC, palbociclib was well tolerated and provided preliminary information on treatment patterns [55].

Combination therapy is a new direction in BC treatment. The purpose of the PALLAS trial (NCT02513394), including 5796 patients, was to determine whether adjuvant endocrine therapy with palbociclib would improve invasive disease-free survival (iDFS) over endocrine therapy alone for HR+/HER2− early breast cancer (EBC) [56]. The most common AEs in the group of patients who received palbociclib and endocrine therapy were neutropenia, leukopenia, fatigue, anemia, alopecia, and upper respiratory tract infection. In addition, they were higher than in the group who received only endocrine therapy. The iDFS at 4 years was 84.2% in the palbociclib plus endocrine therapy group compared to 84.5% in the endocrine therapy alone group (HR 0.96, 95% CI 0.81–1.14; *p* = 0.65). Final analyses showed that the addition of palbociclib to endocrine therapy did not improve outcomes in patients with HR+/HER2− EBC [56,57]. The phase II GEICAM/2014-12 study (FLIPPER, NCT02690480) evaluated the combination of palbociclib and fulvestrant as a first-line therapy in postmenopausal women with HR+/HER2− ABC who had “de novo” metastatic illness or who relapsed after more than 12 months of adjuvant endocrine therapy. The results were better in the palbociclib plus fulvestrant group compared to the placebo plus fulvestrant group. The median PFS was 31.8 versus 22.0 months (HR 0.48; 80% CI 0.37–0.64; *p* = 0.001), and 1-year PFS rates were 83.5% and 71.9% (HR 0.55; 80% CI 0.36–0.83; *p* = 0.064), respectively. Grade ≥ 3 AEs were reported in 80.9% of patients in the palbociclib plus fulvestrant arm and 37.9% in the placebo plus fulvestrant arm [58]. Additionally, the phase II PARFISAL trial (NCT02491983) compared combination therapies, palbociclib plus fulvestrant or letrozole, in patients with HR+/HER2− locally ABC or MBC. The results did not show statistically improvements in PFS (27.9 months vs. 32.8 months; HR = 1.13; 95% CI, 0.89–1.45; *p* = 0.32), objective response rate (ORR) (46.5% vs. 50.2%), or 3-year OS rate (79.4% vs. 77.1%) in the palbociclib plus fulvestrant and palbociclib plus letrozole groups, respectively [59]; however, both combinations confirmed the favorable safety profile [60]. According to those results, more research should be conducted to verify the efficacy and safety of the palbociclib plus endocrine therapy (ET) combination.

The PENELOPE-B study (NCT01864746) was designed for a group of patients with HR+/HER2− BC who have a high risk of relapse after neoadjuvant taxane-containing chemotherapy (NACT). The clinical and pathologic stage (CPS) and estrogen receptor status and histologic grade (EG) system leads to making a valuation of prognosis after NACT. A grade higher than or equal to 3 or 2 and ypN+ was required for inclusion in the study. Patients were divided into placebo plus ET and palbociclib plus ET groups. The iDFS had not been improved by palbociclib: 81.2% in the palbociclib group versus 77.7% in the placebo group (HR 0.93 (0.74–1.17), log-rank *p* = 0.525) [61,62]. However, the subgroup analyses of premenopausal patients who received tamoxifen plus ovarian function suppression (OFS) revealed that the addition of palbociclib resulted in favorable 3-year iDFS versus placebo plus tamoxifen plus OFS (83.0% vs. 74.1%; *p* = 0.053). Furthermore, palbociclib did not influence ovarian function [63].

According to these trials, the toxicity of palbociclib reveals itself in hematological and gastrointestinal side effects, but the efficiency of reducing dosages of palbociclib has not been explored precisely. The Dutch Institute for Clinical Auditing (DICA) medicines program included groups of 598 patients with a median age of 64 years with palbociclib treatment for advanced BC. A total of 33% of them required a dose reduction of palbociclib, and it had more often happened in older patients (≥70 years old). Finally, the OS was similar to the OS of younger patients (20.7 vs. 26.7 months, *p* = 0.051), but the time to next treatment (TTNT) was longer in older patients (16.9 vs. 11.6 months, *p* = 0.013). Additionally, patients with reduced doses of palbociclib had higher OS (29.7 months vs. 21.9 months, *p* = 0.003) and TTNF (16.9 months vs. 11.4 months, *p* < 0.001) versus patients without dose reduction [64]. Treatment with lower doses of palbociclib should be taken into consideration in the group of older patients who are prone to more adverse effects.

The efficiency of palbociclib is being explored in other clinical trials for treating HR+/HER2− MBC. During the phase III of the study PEARL (NCT02028507), postmenopausal women with HR+/HER− MBC who were resistant to prior AI treatment were included in a randomized trial. In the first cohort study, patients received palbociclib plus exemestane versus capecitabine. The second cohort received palbociclib plus fulvestrant versus capecitabine [65]. The results showed that palbociclib plus endocrine therapy did not improve the PFS (7.4 months in the palbociclib plus ET arm vs. 9.4 months in the capecitabine arm) or OS (32.6 months for palbociclib plus ET vs. 30.9 months for capecitabine; *p* = 0.995), but toleration and quality of life were better in this group [66,67]. In the phase I PASTOR study (NCT02599714), postmenopausal patients with ER+/HER2− locally advanced ABC or MBC previously treated by hormonal therapy were divided into two treatment arms of a randomized trial. In this trial, the combination of vistusertib (AZD2012) plus palbociclib plus fulvestrant versus placebo plus palbociclib plus fulvestrant was measured. Patients will be assessed by PFS [68,69].

## 8. Ribociclib

In March 2017, the FDA approved ribociclib [70], a CDK4/6 inhibitor similar to palbociclib in cell cycle inhibition [26]. In the phase II of the MONALEESA-1 trial (NCT01919229), postmenopausal women diagnosed with ER+/HER2− EBC were treated using ribociclib with letrozole or letrozole alone. The combination was well tolerated, with no 3 or 4 grade AEs [71]. The MONALEESA-2 trial (NCT01958021) was designed for postmenopausal HR+/HER2− ABC patients who did not receive prior therapy. The first group received ribociclib plus letrozole, while the second one received placebo plus letrozole, the result being the former group having prolonged PFS (19.3 months vs. 14.7 months; HR 0.556; 95% CI 0.43–0.72; *p* = 0.000000329) [72]. The MONALEESA-3 trial (NCT02422615) evaluated the combination of ribociclib or placebo with fulvestrant in postmenopausal women and men with HR+/HER2− ABC who previously had undergone no or one line of prior endocrine therapy. The PFS was significantly improved in the ribociclib plus fulvestrant group (20.5 months vs. 12.8 months; HR 0.59; 95% CI 0.48–0.73; *p* = 0.00000041) [73]. In the updated analysis, at the data cut-off (30 October 2020), the median OS was 53.7 months in the ribociclib group versus 41.5 months in the placebo group [74]. Across these trials, AEs resembled those of palbociclib, though ribociclib more often caused alanine aminotransferase (ALT) and aspartate aminotransferase (AST) elevation [39]. In the MONALEESA 2 trial, QT prolongation was observed, which led to dose modifications in patients and monitoring the ECG in the first weeks of ribociclib therapy [75].

Phase III of the MONALEESA-7 trial (NCT02278120) was designed for premenopausal patients aged 18–59 years with HR+/HER2− ABC who were treated with goserelin and tamoxifen or goserelin and non-steroidal aromatase inhibitor (NSAI) with or without ribociclib. The PFS was significantly improved in the group with ribociclib (23.8 months vs. 13.0 months; *p* < 0.0000001) [76,77,78]. Common AEs were neutropenia, leukopenia, increased ALT, increased AST, anemia, nausea, and diarrhea [77,78] The 53.5 months of median follow-up confirmed the improvement in OS (58.7 vs. 48.0 months) and PFS (44.2 vs. 31.0 months) for ribociclib plus ET, including patients less than 40 years of age (OS: 51.3 vs. 40.5 months) [79].

The overall findings from MONALEESA confirm the safety profile of adding ribociclib to treatment with fulvestrant or AI in pre-, peri-, and postmenopausal women and men. The AEs were consistent in these trials, and hematologic complications continue to represent the most common grade 3 or 4 AEs [80]. Pooled analysis examining ribociclib dose reduction in patients who developed AEs in the MONALEESA-2, -3, and -7 trials was conducted. A total of 45.8% of patients required ≥1 dose reduction due to AEs; however, 68.5% required only single reduction. For patients who received ribociclib at a relative dose intensity of ≤71% (30th percentile), 72–96% (60th percentile), and 97–100% (90th percentile), the median PFS was 24.8, 24.9, and 29.6 months, respectively [81]. As with the DICA medicines program, this may suggest that dose reduction in older adults will not impact on the quality of ribociclib treatment if modifications are made in compliance with prescribing information.

The purpose of the phase IIIb COMPLEEMENT-1 trial (NCT02941926) was to collect additional safety and efficiency data for the ribociclib plus letrozole combination in pre- and postmenopausal women or men with HR+/HER2− ABC with no prior hormonal treatment [82]. Of the 3246 participants, most experienced treatment-related AEs in all grades (95.2%). The most common were neutropenia (74.5%), increased ALT (16.2%), increased AST (14.1%), and QTc prolongation (6.7%) [83]. Treatment-related AEs grade ≥ 3 occurred in 67.5% of participants, and of treatment-related serious AEs (SAEs) of all grades, 6.5% of SAEs were defined as fatal or life-threatening [82]. The subgroup of 39 men in this trial experienced lesser treatment-related AEs and treatment-related SAEs in comparison with the overall population. Additionally, the incidence of grade ≥ 3 neutropenia in males was lower than in all participants (41% vs. 57.2%), and the overall response was similar to the overall population [84]. Results confirmed the safety profile of ribociclib in combination with letrozole as a first-line treatment for HR+/HER2– ABC in female and male patients [83,84].

The usage of ribociclib has also been investigated in the treatment of EBC. The preliminary safety and tolerability of ribociclib with endocrine therapy as an adjuvant treatment in patients with HR+/HER2− high risk EBC was evaluated in phase 2 of the EarLEE-1 trial (NCT03078751) [85]. Moreover, in the phase 3 NATALEE study (NCT03701334), ribociclib was also tested with or without NSAI as an adjuvant treatment for HR+/HER2− stage II or III EBC. At the data cutoff (11 January, 2023) 3-year iDFS was 90.4% in ribociclib plus NSAI versus 87.1% in the NSAI alone (HR 0.75; 95% CI 0.62–0.91; *p* = 0.003). Additionally, distance disease-free survival and recurrence-free survival also favored the combination [86]. AEs were consistent across age groups. Moreover, the rate of ribociclib discontinuation without dose reduction in older patients demonstrates a chance to improve AE management [87].

## 9. Abemaciclib

The affinity for CDK4 and CDK6 differs in abemaciclib in comparison to palbociclib and ribociclib because of the binding manner. Abemaciclib has a higher binding affinity for the ATP cleft, where it forms a hydrogen bond with Lys43. It is less specific than the others, and it may inhibit CDK1, CDK2, CDK5, CDK9, CDK14, and CDKs16-18 [88]. Ribociclib and palbociclib have a higher affinity for CDK4/6 in contrast with other CDKs due to greater lipophilicity and larger binding site side chains [89,90].

The FDA approved using abemaciclib in combination with fulvestrant for women with HR+/HER2− ABC and MBC with progression after ET in September 2017. Moreover, it was approved as a monotherapy for patients, including men, who had progressed after endocrine therapy and prior chemotherapy in a metastatic setting based on the results from phase II study MONARCH-1 (NCT02102490) [91]. After 12 months of follow-up, the median PFS was 6 months, and the median OS reached 22.32 months. Serious AEs were reported in 33/132 patients (25%). The most common AEs all-grades were diarrhea (90.9%), fatigue, nausea, decreased appetite, neutropenia, and anemia. Most patients (72.3%) experienced grade 2 or 3 diarrhea during their first cycle of medication. They were given anti-diarrheal drugs, which allowed them to avoid reducing their doses or discontinuing treatment [92,93]. The neoMONARCH trial (NCT02441946) compared the application of abemaciclib plus anastrozole versus abemaciclib monotherapy in postmenopausal women with HR+/HER2− EBC. The patients were divided into three groups: abemaciclib plus anastrozole (Ab+An), abemaciclib monotherapy (Ab), and anastrozole (An) monotherapy. The primary objective evaluated change in Ki67 expression from baseline after 2 weeks of treatment, which was greater in the two abemaciclib arms: Ab + An—(−93%), Ab—(−91%), An—(−63%). Moreover, complete cell-cycle arrest, defined as Ki67 ≤ 2.7%, was achieved in more patients treated with the abemaciclib (Ab + An—68%, Ab—58%, An—14%; *p* < 0.001) [75,94,95].

The next trials evaluated the efficacy and safety of abemaciclib in combination with endocrine therapy. The phase III study MONARCH-2 (NCT02107703) compared the PFS in two groups of women with HR+/HER2− ABC. The first group received abemaciclib plus fulvestrant, and the second received fulvestrant in monotherapy. The PFS was higher in the abemaciclib arm (16.4 months vs. 9.3 months; *p* < 0.0000001). The most common AEs were diarrhea (87.07%), neutropenia (49.66%), nausea 49.43%), fatigue (43.54%), and anemia (35.15%). Diarrhea of grade 1 or 2 occurred more frequently than grade 3 diarrhea, but the majority of patients did not require dose modification [39,96,97]. The phase III study MONARCH-3 (NCT02246621) investigated the usage of abemaciclib in combination with a NSAI in postmenopausal women with HR+/HER2− ABC with no prior systemic therapy. Finally, median OS was higher in the abemaciclib plus NSAI group, at 66.8 months versus 53.7 months, and 63.7 months versus 48.8 months in the subgroup with visceral disease. Statistical significance was not attained (*p* = 0.0664 and *p* = 0.0757, respectively), but the results were comparable with the 12.5 months improvement in median OS in the MONALEESA-2 trial. Administering abemaciclib in addition to NSAI resulted in a significantly improved PFS (29 vs. 14.8 months; *p* < 0.0001) [98]. The most common grade 3 or 4 AEs were neutropenia, diarrhea, and leukopenia. All other grade AEs were diarrhea, mainly grade 1, anemia, abdominal pain, fatigue, and nausea [99,100].

Within the first ten years, even 20% of patients with HR+/HER2 may have a disease recurrence [101]. The MONARCH-2 and MONARCH-3 trials showed that adding abemaciclib into therapy significantly improved the PFS and OS, so the purpose of the phase III MonarchE trial (NCT03155997) was to investigate the addition of this CDK4/6 inhibitor to standard adjuvant ET in patients with HR+/HER2−, node-positive, high-risk EBC [101,102]. From July 2017 to August 2019, a group of 5637 patients were assigned to the trial, and after the cutoff in March 2020, a 2-year treatment period had been completed in 707 patients. The addition of abemaciclib to ET improved the iDFS (92.2% vs. 88.7%; *p* = 0.01) and distant relapse-free survival (DRFS) (93.6% vs. 90.3%; HR 0.72, 95% CI 0.56–0.92; *p* = 0.01) in comparison to ET alone [101]. After 5 years, the iDFS and DRFS difference totaled 7.6%, and 6.7%, respectively, and it was higher in the abemaciclib arm [103]. This trial showed the impact of abemaciclib in reducing the risk of early metastatic recurrence [101]. Further analysis is needed to determine whether the OS can be enhanced [104]. Jiang et al. evaluated the efficacy and safety of abemaciclib with switching ET versus chemotherapy in Chinese patients who progressed on prior palbociclib plus ET. The results showed that the median PFS was longer in the abemaciclib group (6 vs. 4 months; *p* = 0.667). Moreover, the PFS was the same in the group of patients who had fewer lines of prior systemic therapy (6 vs. 6 months); however, it was significantly higher in the group of patients who were treated with palbociclib as a first-line therapy in comparison to prior palbociclib as ≥second-line therapy (11.0 vs. 5.0 months; *p* = 0.043). The results suggest that abemaciclib with switching ET could be a possible course of treatment for Chinese individuals with HR+/HER2− MBC [105].

## 10. Dalpiciclib

Dalpiciclib (SHR6390) is a newer CDK4/6 inhibitor that interacts with the ATP-bonding cleft in the same way as palbociclib, ribociclib, and abemaciclib. It has shown antitumor activity in esophageal squamous cell carcinoma and RB-positive cancer cell lines by blocking the progression of the cell cycle from the G1 phase to the S phase [106,107]. Moreover, it overcame acquired drug resistance to trastuzumab and tamoxifen in BC [107]. The aim of the phase III DAWNA-1 study (NCT03927456) was to evaluate the safety and efficacy of dalpiciclib with or without fulvestrant in HR+/HER− recurrent or metastatic BC patients who had prior endocrine therapy. The PFS was significantly greater in the dalpiciclib plus fulvestrant combination (15.7 vs. 7.2; one-sided *p* < 0.0001). The most common 3 or 4 grade AEs were neutropenia (84.2%) and leukopenia (62.1%). The most common non-hematological low-grade AEs were liver enzyme abnormalities [108,109]. In the phase III DAWNA-II trial (NCT03966898), patients with HR+/HER2− recurrent or metastatic BC with no prior systemic therapy were randomly assigned (2:1) to receive dalpiciclib plus ET (letrozole or anastrozole) or placebo plus ET. At data cutoff (1 June 2022), median PFS was significantly longer in the dalpiciblib plus ET than in the placebo group (30.6 vs. 18.2 months; one-sided log-rank *p* < 0.0001). Grade 3 or 4 AEs were reported more frequently in the dalpiciclib group (90% vs. 12%), where the most common were neutropenia and leukopenia [110]. The results from the DAWNA-1 and DAWNA-2 trials show that dalpiciclib is a viable new treatment option for relapsing or progressing HR+/HER2− BC (Table 3).

## 11. Continuation of CDK4/6 Inhibitors in Second Line After Prior Exposure

Disease progression results from the development of resistance to CDK4/6i therapy over time, making it difficult to choose the best course of treatment. Considering optimal quality of life and a tolerable toxicity profile some of patients can switch to another CDK4/6i, but ET in combination with targeted therapy should prioritize [111]. The results from the TRINITI-1 trial (NCT02732119) indicated that co-targeting of the PI3K/mTOR signaling pathway using mTOR inhibitors and blocking CDK4/6 in combination with aromatase inhibitors warrants further investigation in patients previously treated with CDK4/6 inhibitors [112]. Due to the fact that mTORC2 can cause resistance to mTOR inhibitors via AKT phosphorylation at a secondary location (Ser473), PI3Kα-specific inhibitors or AKT inhibitors, like alpelisib and capivasertib, respectively, may be an option to break the resistance [113]. Currently, alpelisib is approved by the FDA for patients with PIK3CA mutation, after prior ET, based on the results from SOLAR-1 trial [114]. In the CAPItello-291 (NCT04305496) trial, capivasertib with fulvestrant showed significantly longer PFS than treatment with fulvestrant alone (7.3 vs. 3.1 months; *p* < 0.001) among patients with HR+/HER2− ABC whose disease had progressed during or after previous AI therapy with or without a CDK4/6 inhibitor [115]. In the phase 1b/3 CAPItello-292 (NCT04862663) trial, capivasertib will be assessed in combination with palbociclib and fulvestrant in patients with HR+/HER2−, locally advanced, unresectable MBC [116].

Targeting *ESR1* gene mutation acquired during CDK4/6i plus AI therapy is the purpose of selective ER degraders (SERDs) like elacestrant, which was approved for the treatment of *ESR1* gene mutation and HR+/HER2− MBC [117]. The results of the phase III EMERALD trial (NCT03778931) demonstrated that combining CDK4/6i with elacestrant was more effective than CDK4/6 inhibitors with ET (median PFS: 3.8 vs. 1.9 months) [118]. Moreover, in the ELEVATE trial (NCT05563220), the activity of elacestrant will be assessed in various combinations (with abemaciclib, capivasertib, everolimus, alpelisib, ribociclib, and palbociclib) in patients with ER+/HER2− ABC or MBC [119]. Other SERDs, such as imlunestrant (NCT04975308), giredestrant (NCT05306340, NCT04802759), camizestrant (NCT03616587), rintodestrant (NCT03455270), ZN-c5 (NCT04514159, NCT03560531), and D-0502 (NCT03471663), are being evaluated in a number of trials to determine their efficacy in combination with CDK4/6i or PI3K/AKT/mTOR inhibitors following the progression of the disease on CDK4/6i [111].

The *ESR1* mutations prompt searching for new combinations of drugs, including a third generation of selective estrogen receptor modulators (SERM). One such agent, lasofoxifene, in the ELAINE (NCT03781063) study, showed encouraging antitumor activity versus fulvestrant among women with locally advanced or metastatic ER+/HER2− BC expressing ERα mutants (median PFS: 5.6 vs. 3.7 months) [120]. Therefore, it was tested in combination with a CDK4/6 inhibitor in the phase II ELAINE 2 (NCT04432454) study. Longer follow-up showed clinically meaningful efficacy and good toleration [121,122]. In patients who progressed on an AI with palbociclib or ribociclib as their initial hormonal treatment, the phase III ELAINE 3 trial (NCT05696626) will now evaluate the effectiveness of lasofoxifene plus abemaciclib for treating locally advanced or metastatic ER+/HER2-BC with an *ESR1* mutation [123].

The use of CDK4/6 inhibitors after progression on previous treatment with this class of drugs has been evaluated in several studies. Following progression on CDK4/6i (mainly palbociclib), the phase II MAINTAIN trial (NCT02632045) showed an improvement in PFS in ribociclib plus switched ET versus placebo plus switched ET (5.29 vs. 2.76 months, respectively; *p* = 0.006) [124]. Moreover, in the phase III postMONARCH trial (NCT05169567), the combination of abemaciclib and fulvestrant statistically significantly improved PFS after disease progression of previous CDK4/6i plus ET, including patients with or without *ESR1* or *PIK3CA* mutations (PFS rates at 6 months of 50% vs. 37% for the abemaciclib and placebo arms, respectively) [125]. However, the results of the PACE (NCT03147287) and PALMIRA (NCT03809988) trials did not show efficacy in treatment with palbociclib plus second-line ET after progression of first-line palbociclib plus ET [126,127]. Notably, in the PACE trial, longer PFS was observed when combining palbociclib with avelumab (PD-L1 inhibitor) [126]. Continuation with different CDK4/6i, rather than the same agent, with switched ET may be an option for patients who progressed after first-line setting [128], but more studies should be conducted to verify the effectiveness and safety of this strategy. Additionally, considering acquired mutations during therapy, other combinations using SERD, SERM, or PI3K/AKT/mTOR inhibitors should be further investigated.

## 12. Recommendations for CDK4/6 Inhibitor Treatments

The National Comprehensive Cancer Network (NCCN) guidelines recommend ribociclib and abemaciclib over palbociclib as adjuvant therapy due to the iDFS improvements demonstrated in phase III trials: NATALEE for ribociclib and monarchE for abemaciclib [129]. In contrast, the final analyses of the PALLAS and PENELOPE-B trials showed that adding palbociclib to adjuvant ET did not improve iDFS compared to ET alone [57,61].

Introducing CDK4/6 inhibitors into a patient’s therapy requires the attending physician to fully assess the patient’s health, including the presence of comorbidities, permanent medication, supplements, diet, and allergies, due to the possible occurrence of an increased number of comorbidities. Choosing the right CDK4/6 inhibitor may be a challenge, so it is important to know the contraindications to their use [130]. Abemaciclib has the shortest half-life. The recommended dosage is 200 mg twice daily in monotherapy or 150 mg twice daily in combination with endocrine therapy, with no break in between. In comparison, palbociclib and ribociclib are administered for 21 days at doses of 125 mg daily and 600 mg daily, followed by 7 days without treatment, which reduces the risk of neutropenia, common in this group of drugs [88,130,131,132]. The conditions of absorption differ among CDK4/6 inhibitors. Palbociclib does not interact with food but its absorption decreases with fasting, while abemaciclib interacts only in its prodrug form, and its absorption increases with a high-fat diet [130,132]. Cytochrome P450 3A4 (CYP3A4) and SULT2A1 are responsible for the hepatic metabolism of these drugs [88,133]. Due to CYP3A4’s extensive metabolism of abemaciclib, it can interact with other CYP3A4 substrates, inducers, and inhibitors [134]. The main route of excretion is through the gastrointestinal tract [88,133]. None of these inhibitors are recommended for pregnant or breastfeeding women. Dose reduction does not decrease the OS rate, so it should be taken into consideration in elderly patients because AEs are more common in patients >75 years of age [130].

Research has shown that palbociclib and ribociclib have higher rates of bone marrow toxicity while abemaciclib is mostly associated with gastrointestinal system problems [81]. In the MONARCH-2 and MONARCH-3, trials more than 80% of patients reported diarrhea as an adverse effect [45,88,130,131]. The incidence of nausea and vomiting was the lowest for the palbociclib treatment; however, it had the highest rate of neutropenia. The risk of febrile neutropenia for any of these three inhibitors remains low (<2%) [130,135]. Ribociclib, in phase III trials, has shown hepatotoxicity with elevated transaminases levels, higher hypertension incidence, and cardiovascular AEs, where corrected QT (QTc) prolongation was the most frequent [130,131]. Ribociclib should be avoided when the patient suffers from cardiological diseases like bradyarrhythmia or long QT syndrome. Recent ischemic myocardial syndrome, heart failure, or electrolyte abnormalities also eliminate patients from therapy with ribociclib [132]. Older patients are exposed to QT prolongation due to increasing age and heart condition. Moreover, everyday medications combined with ribociclib could lead to QT prolongation and torsade de pointes, which could be fatal, so it is important to consider AEs during the selection of the treatment [130,133].

During therapy, it is recommended to monitor the patient’s health by performing a complete blood count for all three drugs, check of liver function tests (LFTs) for ribociclib and abemaciclib, and monitoring of QTc interval along with electrocardiogram (ECG) for ribociclib. It is extremely important that in the case of prolonged QTc > 500 ms, the physician should consider interrupting treatment until it declines to <481 ms [81]. To avoid these occurrences, it is crucial to investigate the cellular and molecular mechanisms by which CDK4/6 inhibitors impact the heart, as well as biomarkers that can predict or detect preliminary signs of cardiotoxicity (Table 4) [136].

## 13. CDK4/6 Inhibitors in HER-Positive Tumors

Approximately 15–20% of BC exhibits amplification or overexpression of the *HER2* gene, which is associated with metastasis and proliferation [137]. Although there are therapies for HER+ BC using monoclonal antibodies, kinase inhibitors, or antibody-drug conjugate (ADC), developed resistance to the treatment becomes incurable [138]. Investigating new treatment options like CDK4/6 inhibitors is a new target for the researchers.

The purpose of the phase II MonarcHER trial (NCT02675231) was to evaluate the efficacy of the combination of abemaciclib plus trastuzumab with (Arm A) or without (Arm B) fulvestrant in comparison to trastuzumab plus standard-of-care (SOC) chemotherapy (Arm C) in women with HR+/HER2+ locally advanced or metastatic BC after prior exposure to a minimum of two HER2-directed therapies for advanced disease [139,140]. The median OS in Arm A, B, and C rose to 31.1, 29.2, and 20.7 months, respectively. The PFS was also the highest in Arm A (8.3 months vs. 5.7 months vs. 5.7 months). The results showed improvement in median OS using abemaciclib plus trastuzumab with or without fulvestrant combination in contradistinction to SOC chemotherapy plus trastuzumab. Additionally, luminal subtypes showed longer OS (31.7 vs. 19.7 months) and PFS (8.6 vs. 5.4 months) than non-luminal subtypes during exploratory analysis [141]. Similarly, in the phase II PATRICIA trial, the combination of palbociclib and trastuzumab demonstrated a synergistic benefit, especially in patients with luminal disease (defined by PAM50, cohorts B1 and B2), who experienced longer PFS compared to those with non-luminal subtypes (PFS: 10.6 vs. 4.2 months, *p* = 0.003) [142]. The ongoing phase III PATINA trial (NCT02947685) aims to determine whether palbociclib plus anti-HER2 therapy plus endocrine therapy (ET) is superior to HER2-targeted therapy plus ET alone in HR+/HER2+ BC by measuring PFS [138,143].

Dalpiciclib (SHR6390) was recently approved for treating advanced BC in China, based on promising results from the phase III DAWNA-1 trial [144]. The DAP-Her-01 study (NCT04293276) is currently evaluating the usage of orally administered dalpiciclib in combination with pyrotinib as a first-line treatment in HER2+ ABC. Preliminary results indicate promising activity. The median PFS was 11 months (95% CI 7.3–19.3, median follow-up of 19.2 months). The most common AEs were leukopenia (68.3%), neutropenia (65.9%), and diarrhea, but most of them were tolerable [145,146]. Additionally, the current trial DAP-HER2-02 (NCT05328440) investigates first-line treatment for HER2+ ABC with a combination of dalpiciclib plus pyrotinib with fulvestrant or inetetamab (cipterbin) (Table 5).

## 14. Raising Concerns and Cost-Effectiveness of CDK4/6 Inhibitors

Haslam et al. re-evaluated the NATALEE and MonarchE trials and questioned the efficacy of the treatment to some extent. Both trials demonstrated modest improvements in iDFS (absolute differences of 7.6% in MonarchE and 3.3% in NATALEE; HR 0.75 and 0.68, respectively), suggesting a potential reduction in recurrence; however, the drop-out rates in the control groups were substantial, which might have distorted the results, favoring the experimental arm. The trials reported minimal OS, leaving the long-term significance of iDFS gains uncertain. In addition, the experimental arms experienced a much higher frequency of grade 3 or higher AEs, and the cost burden remains high. Unless additional placebo-controlled trials are conducted or patients who are more likely to benefit are better categorized, the article suggests that these treatments for ER+/HER2− EBC should not become commonplace [147].

Given the uncertain benefit in efficacy of adding CDK 4/6 to first- rather than second-line endocrine treatment, the aim of the phase III SONIA trial (NCT03425838) was to evaluate whether the sequence of an aromatase inhibitor plus CDK 4/6 in first line followed by fulvestrant in second line is superior to the sequence of an aromatase inhibitor in first line followed by fulvestrant plus CDK4/6 in second line for patients with HR+/HER2− ABC [148]. The time from randomization to disease progression after second-line treatment was measured, and there was no statistically significant benefit for the use of CDK4/6i as a first-line treatment (31.0 versus 26.8 months, respectively; HR 0.87; 95% CI 0.74–1.03; *p* = 0.10). Additionally, the treatment duration was longer in the first-line CDK4/6i group (24.6 versus 8.1 months), and more grade ≥ 3 AEs were reported. This trial suggests that second-line therapy with CDK4/6i may be preferred option [149].

A thorough examination of the financial implications of CDK4/6i therapy and its accessibility in various healthcare systems is particularly important considering the large number of research on CDK4/6i and its proven effectiveness in treatment. Based on a meta-analysis and systematic review of 16 and 18 articles, respectively, it was shown that CDK4/6 inhibitors were not cost-effective compared with hormone therapy/aromatase inhibitors alone in postmenopausal women. The incremental net benefit (INB) was negative: USD −149,266.87, especially in the USA and China. The authors suggest that lowering drug prices or finding alternative treatment options may improve the situation. The need to describe the economic properties of CDK4/6i for different subgroups, such as patients receiving second-line treatment and non-postmenopausal women, was highlighted [150].

Based on data from the SONIA trial, adding CDK4/6i to second-line hormone therapy instead of first-line therapy in patients with ABC is associated with per-patient costs, but overall healthcare expenditure is significantly reduced. This may be an option to increase INB [151]. Moreover, according to the data from the PALOMA-2, MONALEESA-2, MONARCH-3, and PO25 studies, abemaciclib was found to be the most cost-effective option of all three CDK4/6 inhibitors. However, when analyzing the quality of life adjusted for life years (QALY), ribociclib was the most effective option from the healthcare payer perspective in Qatar [152]. In the analysis of data from the MONALEESA-7 study, it was shown that the combination of ET with ribociclib was more cost-effective than endocrine therapy alone [153]. In turn, from the perspective of payers in China, abemaciclib plus ET was a cost-effective treatment option compared with placebo plus ET [154]. In summary, the cost-effectiveness of CDK4/6 inhibitors may depend on the price, the strategy of use, and the healthcare system in each country. Further cost-effectiveness analyses are needed to optimize the use of these drugs and improve the accessibility of the therapy.

## 15. Conclusions

CDK4/6 inhibitors can inhibit the cell cycle by suppressing the G1 to S transition. Each of them is slightly different in its effect, thanks to which the possibilities of their use are constantly expanding. Primarily, in combination with AIs, the PALOMA-2, MONALEESA-2, and MONARCH-3 studies demonstrated improved PFS in postmenopausal women. As a result of those studies, palbociclib, ribociclib, and abemaciclib have been approved for use in first- and later lines of therapy in women with HR+HER2− MBC regardless of age or endocrine sensitivity. Moreover, the analysis of premenopausal and perimenopausal women in the MONALEESA-7 study showed promising results, where PFS increased statistically significantly.

In the PALOMA-3, MONALEESA-3, and MONARCH-2 trials, the combination with the CDK4/6 inhibitor showed improvement in PFS in patients who progressed on prior endocrine therapy. Although palbociclib showed no benefit in adjuvant therapy, abemaciclib and ribociclib showed improvement in iDFS in the MonarchE and NATALEE studies, respectively. The effects of CDK4/6 inhibitors have been studied in many different combinations. At this moment, only abemaciclib is approved by the FDA as monotherapy for MBC. Further research of CDK4/6 inhibitors application is ongoing, including, for example, the recent approval of dalpiciclib in the treatment of HR+/HER+ BC.

The toxicity profile of CDK4/6 inhibitors is similar. The most common side effects include neutropenia, leukopenia, fatigue, diarrhea, and nausea. Ribociclib may additionally cause QTc prolongation and abemaciclib may cause grade 3 diarrhea. Compared with chemotherapy, which has a deleterious effect on healthy cells, CDK4/6 inhibitors make a better treatment option when endocrine therapy does not elicit a response. The phase III PEARL trial (NCT02028507) analysis showed that inhibitors combined with endocrine therapy versus chemotherapy did not affect PFS in patients. Moreover, the combination with inhibitors improved the quality of life, tolerability, and toxicity profile and reduced time to deterioration of global health status [155]. For this reason, CDK4/6 inhibitors are being used more frequently to treat HR+/HER2− breast cancer. A deeper understanding of their action will allow for even more effective treatment of breast cancer, not only HR+/HER2− but also other types.

## Figures and Tables

**Table 1:** Molecular subtypes of breast cancer with IHC phenotype, histologic subtypes, and metabolic difference.

Subtype	Luminal A	Luminal B	HER2-Enriched	Basal-like/TNBC
IHC Phenotype	ER+ HER2−	ER+ HER2−	ER- HER2+	ER- HER2−
	PR ≥ 20% Ki67 < 20%	And/or PR < 20% and/or Ki67 ≥ 20%	PR-	PR-
DNA mutations	TP53 (12%) PIK3CA (49%)	TP53 (32%) PIK3CA (32%)	TP53 (84%) PIK3CA (7%)	TP53 (75%) PIK3CA (42%)
Glucose metabolism	Lower than HER-enriched Mainly TCA cycle, reverse Warburg effect	Lower than HER-enriched Mainly TCA cycle, reverse Warburg effect	Higher than luminal A and B Mixed metabolism—glycolysis and TCA	The highest glucose metabolism activity Mainly glycolysis, Warburg effect The highest level of GLUT1 expression
Amino acid metabolism	Lower than in HER-enriched	Lower than in HER-enriched	The highest	The lowest
Lipid metabolism	Lower than in HER-enriched	Lower than in HER-enriched	The highest	The lowest
Glutamine metabolism	Lower than in luminal B	Higher than in luminal A	The highest	-

Abbreviations: IHC—immunohistochemical; ER—estrogen receptor; PR—progesterone receptor; HER2—human epidermal growth factor receptor 2; TCA—tricarboxylic acid cycle; GLUT—glucose transporter; PIK3CA—phosphatidylinositol-4,5-bisphosphate 3-kinase catalytic subunit alpha; TNBC—triple negative breast cancer.

**Table 2:** CTCAE v5.0 for the most frequent adverse events occurring during breast cancer treatment with CDK4/6 inhibitors [41].

CTCAE Term	Grade 1	Grade 2	Grade 3	Grade 4	Grade 5
Diarrhea	<4 stools per day, Mild increase in ostomy output	4–6 stools per day, Moderate increase in ostomy output, limiting instrumental ADL	≥7 stools per day, Severe increase in ostomy output, limiting self-care ADL	Life-threatening consequences, urgent intervention indicated	Death
Neutropenia	<LLN—1500/mm^3^; <LLN—1.5 × 10^−9^/L	<1500–1000/mm^3^; <1.5–1.0 × 10^−9^/L	<1000–500/mm^3^; <1.0–0.5 × 10^−9^/L	<500/mm^3^; <0.5 × 10^−9^/L	-
Leukopenia	<LLN—3000/mm^3^; <LLN—3.0 × 10^−9^/L	<3000–2000/mm^3^; <3.0–2.0 × 10^−9^/L	<2000–1000 /mm^3^; <2.0–1.0 × 10^−9^ /L	<1000/mm^3^; <1.0 × 10^−9^/L	-
Thrombocytopenia	<LLN—75,000/mm^3^; <LLN—75.0 × 10^−9^/L	<75,000–50,000/mm^3^; <75.0–50.0 × 10^−9^/L	<50,000–25,000 /mm^3^; <50.0–25.0 × 10^−9^ /L	<25,000/mm^3^; <25.0 × 10^−9^/L	-
Anemia	Hgb <LLN—10.0 g/dL; <LLN—6.2 mmol/L; <LLN—100 g/L	Hgb <10.0–8.0 g/dL; <6.2–4.9 mmol/L; <100–80 g/L	Hgb <8.0 g/dL; <4.9 mmol/L; <80 g/L; transfusion indicated	Life-threatening consequences, urgent intervention indicated	Death

Abbreviations: CTCAE—Common Terminology Criteria for Adverse Events; ADL—Activities of Daily Living; Hgb—hemoglobin; LLN—Lower Limits of Normal.

**Table 3:** The trials with palbociclib, ribociclib, abemaciclib, and dalpiciclib.

Stage of Breast Cancer, Type of Therapy (Adjuvant/Neoadjuvant)	Drug	Study	Phase	Population and Menopausal Status	Number of Participants	Primary or Secondary Endpoints 1. With Inhibitor 2. Without Inhibitor	Most Common AEs
EBC Adjuvant	PALBOCICLIB	PALLAS (NCT02513394)	III	ER+/HER2− EBC	5796	iDFS (4 years) 1. 84.2% 2. 84.5%	Neutropenia, leukopenia, fatigue, anemia, alopecia, upper respiratory tract infection
EBC Adjuvant	PALBOCICLIB	PENELOPE-B (NCT01864746)	III	HR +/HER2− normal primary BC but high relapse risk after neoadjuvant chemotherapy	1250	iDFS (3 years) 1. 81.2% 2. 77.7%	Leukopenia, neutropenia, anemia, thrombocytopenia, febrile neutropenia, fatigue, nausea, stomatitis
EBC	RIBOCICLIB	MONALEESA-1 (NCT01919229)	II	HR+/HER2− EBC postmenopausal	14	Mean decrease in Ki67-expressing cells, 1. 96% or 92%, 2. 69%	Nausea, decreased appetite, diarrhea, abdominal pain, fatigue, asthenia
EBC Adjuvant	RIBOCICLIB	EarLEE-1 (NCT03078751)	II	HR+/HER2− high risk EBC	54	% of AEs and SAEs 1. Aes—96.2%, SAEs—15.4% 2. AEs—87.5%, SAEs—8.3%	Neutropenia, anemia, thrombocytopenia, diarrhea, nausea, fatigue, increased ALT, increased AST SAEs: disseminated intravascular coagulation, AML, pulmonary embolism, cellulitis, cardiac failure congestive
EBC Adjuvant	RIBOCICLIB	NATALEE (NCT03701334) Data cutoff 11 January 2023	III	HR+/HER2− EBC	5101	3-y iDFS 1. 90.4% 2. 87.1%	Neutropenia, arthralgia, fatigue, nausea
EBC Adjuvant	ABEMACICLIB	MonarchE (NCT03155997)	III	HR+/HER2− High-risk, node-positive, early stage surgery of the primary	5637	4-y iDFS 1. 85.8% 2. 79.4%	Leukopenia, neutropenia, anemia, thrombocytopenia, fatigue, nausea, abdominal pain
EBC Neoadjuvant	ABEMACICLIB	NeoMONARCH (NCT02441946)	II	HR+/HER2− EBC postmenopausal	224	Percent change in Ki67 expression (from baseline to 2 weeks) 1. Ab + An (−93%) 2. Ab (−91%) 3. An (−63%)	Diarrhea, nausea, fatigue, constipation
ABC/MBC	PALBOCICLIB	PALOMA-1/TRIO-18 (NCT00721409)	II	ER+/HER2− ABC postmenopausal	177	PFS 1. 20.2 months 2. 10.2 months	Neutropenia, leukopenia, fatigue anemia, nausea, backpain
ABC/MBC	PALBOCICLIB	PALOMA-2 (NCT01740427)	III	ER+/HER2− ABC postmenopausal	666	PFS 1. 19.3 months 2. 12.9 months	Neutropenia, leukopenia, nausea, fatigue, arthralgia, alopecia
ABC/MBC	PALBOCICLIB	PASTOR (NCT02599714)	I	ER+/HER2− locally advanced or MBC prior hormonal therapy postmenopausal	54	Number of AEs—parts A and B PFS—part B	No results posted
ABC/MBC	PALBOCICLIB	PARFISAL (NCT02491983)	II	ER+/HER2− locally advanced or MBC	486	PFS (median follow-up of 32 months) 1. palbociclib + letrozole—32.8 months 2. palbociclib + fulvestrant—27.9 months	Neutropenia, leukopenia, anemia, asthenia, arthralgia, fatigue, diarrhea
ABC/MBC	RIBOCICLIB	MONALEESA-2 (NCT01958021)	III	HR+/HER2− MBC without prior therapy postmenopausal	668	PFS 1. 19.3 months 2. 14.7 months	Neutropenia, leukopenia, nausea, fatigue, diarrhea, alopecia, increased ALT, increased AST
ABC/MBC	RIBOCICLIB	MONALEESA-3 (NCT02422615)	III	HR+/HER2− ABC without or one line prior endocrine therapy postmenopausal	726	PFS 1. 20.5 months 2. 12.8 months OS (data cut-off—30 October 2020) 1. 53.7 months 2. 41.5 months	Neutropenia, leukopenia, nausea, fatigue, diarrhea, alopecia, rash, arthralgia, anemia, increased ALT, increased AST
ABC/MBC	RIBOCICLIB	MONALEESA-7 (NCT02278120)	III	ER+/HER2− advanced MBC premenopausal or perimenopausal	672	PFS 1. 23.8 months 2. 13.0 months	Neutropenia, leukopenia, increased ALT, increased AST, anemia, nausea, diarrhea, hypertension
ABC/MBC	ABEMACICLIB	MONARCH-1 (NCT02102490)	II	HR+/HER2− MBC or ABC prior treatment with at least two chemotherapy regimens	132	PFS 6.0 months (95% confidence interval (CI) 4.2 to 7.5)	Diarrhea, fatigue, nausea, decreased appetite, abdominal pain, thrombocytopenia, neutropenia,
ABC/MBC	ABEMACICLIB	MONARCH-3 (NCT02246621)	III	HR+/HER2− MBC, recurrent or locoregionally postmenopausal	493	PFS 1. 29.0 months 2. 14.8 months Final mOS 1. 66.8 months 2. 53.7 months	Neutropenia, diarrhea, nausea, fatigue, infections
ABC/MBC No prior systemic therapy	DALPICICLIB	DAWNA-2 (NCT03966898) Data cutoff 1 June 2022	III	HR+/HER2− MBC or recurrent BC	426	PFS 1. 30.6 months 2. 18.2 months	Neutropenia, leukopenia
ABC/MBC prior endocrine therapy	PALBOCICLIB	PALOMA-3 (NCT01942135)	III	HR+/HER2− MBC prior endocrine therapy	521	PFS 1. 9.2 months 2. 3.8 months OS 1. 34.8 months 2. 28.0 months	Neutropenia, leukopenia, fatigue, nausea, headache, alopecia
ABC/MBC prior endocrine therapy	ABEMACICLIB	MONARCH-2 (NCT02107703)	III	HR+/HER2− MBC or local ABC prior endocrine therapy	669	PFS 1. 16.4 months 2. 9.3 months	Neutropenia, diarrhea, nausea, fatigue, abdominal pain
ABC/MBC prior endocrine therapy	DALPICICLIB	DAWNA-1 (NCT03927456)	III	HR+/HER2− MBC or recurrent BC prior endocrine therapy	361	PFS 1. 15.7 months 2. 7.2 months	Neutropenia, leukopenia, thrombocytopenia, prolonged OT, liver enzyme abnormalities
ABC/MBC CDK4/6 inhibitor resistance	RIBOCICLIB	TRINITI-1 (NCT02732119)	I/II	HR+/HER2− locally advanced or MBC Progression on a CDK4/6 inhibitor Group 1: 300 mg ribociclib + 2.5 mg everolimus +25 mg exemestane Group 2: 200 mg ribociclib + 5 mg everolimus + 25 mg exemestane	104	CBR—phase II 1. 65.2% 2. 59.4%	Neutropenia, anemia, thrombocytopenia, stomatitis, diarrhea
Special populations	PALBOCICLIB	PEARL (NCT02028507)	III	HR+/HER2 MBC resistance to aromatase inhibitors postmenopausal	693	PFS 1. 7.4 months 2. 9.4 months OS 1. 32.6 months 2. 30.9 months	Neutropenia, leukopenia, hand/foot syndrome, diarrhea, fatigue
Special populations Neoadjuvant	PALBOCICLIB	NeoPalAna (NCT01723774)	II	ER+/HER2− stage 2 or 3 Arm 1—PIK3CA wild type Arm 2—PIK3CA mutant type Arm 3—endocrine resistant	50	Ki67 ≤ 2.7% (complete cell arrest) following 2 weeks 1. 79.3% 2. 100% 3. 57.6%	Neutropenia, leukopenia, thrombocytopenia, anemia, nausea, headache, arthralgia, diarrhea
Special populations	PALBOCICLIB	FLIPPER (NCT02690480)	II	HR+/HER2− MBC Postmenopausal de novo metastatic disease or prior ET ≥ 5 years and remained disease free for >12 months	189	PFS 1. 31.8 months 2. 22.0 months 1-y PFS 1. 83.5% 2. 71.9%	Neutropenia, anemia, leukopenia, thrombocytopenia, fatigue, diarrhea
Special populations	RIBOCICLIB	PATINA (NCT02947685)	III	HR+/HER2+ MBC	496	PFS	No results posted

Abbreviations: ER—estrogen receptor; HER2—human epidermal growth factor 2; ABC—advanced breast cancer; MBC—metastatic breast cancer; EBC—early breast cancer; ET—endocrine therapy; AEs—adverse events; SAEs—serious adverse events; ALT—alanine aminotransferase; AST—aspartate aminotransferase; CBR—clinical benefit rate; iDFS—invasive disease-free survival; Ab—abemaciclib; An—anastrozole.

**Table 4:** Adverse effects differences between palbociclib, ribociclib, and abemaciclib.

	Palbociclib	Ribociclib	Abemaciclib
Hematologic toxicity, especially neutropenia	+++	++	+
Gastrointestinal toxicity, including diarrhea	+	+	+++
Cardiotoxicity, including QT prolongation	−	++	−

Abbreviations: “+”—mild; “++”—moderate; “+++”—severe; “−”—not significant or not observed.

**Table 5:** Clinical trials for CDK4/6 inhibitors in HER-positive tumors.

Trial	Phase	Patients	Number of Patients	Treatment	Results	Adverse Events
DAP-HER-01 (NCT04293276)	II	HER2+ ABC ≤1 prior endocrine therapy	41	Dalpiciclib + pyrotinib	mPFS in evaluable patients: 11.0 months	Leukopenia, neutropenia, diarrhea
DAP-HER-02 (NCT05328440)	II	HR+/HER2+ ABC	120	Dalpiciclib + pyrotinib + fulvestrant/inetetamab	PFS	No results posted
MonarcHER (NCT02675231)	II	HR+/HER2+ locally advanced or MBC ≥2 prior HER2-directed therapies	237	Arm A abemaciclib + trastuzumab + fulvestrant Arm B abemaciclib + trastuzumab Arm C trastuzumab + SOC therapy	PFS 1. 8.3 months 2. 5.7 months 3. 5.7 months	Neutropenia, diarrhea, anemia, nausea, fatigue, abdominal pain
PATINA (NCT02947685)	III	HR+/HER2+ MBC	496	Palbociclib + trastuzumab/pertuzumab + endocrine therapy	PFS	No results posted

Abbreviations: HR—hormone receptor; HER2—human epidermal growth factor 2; ABC—advanced breast cancer; MBC—metastatic breast cancer; mPFS—(median) progression-free survival; SOC—standard of care.
